# Dietary Fat Content and Fiber Type Modulate Hind Gut Microbial Community and Metabolic Markers in the Pig

**DOI:** 10.1371/journal.pone.0059581

**Published:** 2013-04-03

**Authors:** Hui Yan, Ramesh Potu, Hang Lu, Vivian Vezzoni de Almeida, Terry Stewart, Darryl Ragland, Arthur Armstrong, Olayiwola Adeola, Cindy H. Nakatsu, Kolapo M. Ajuwon

**Affiliations:** 1 Department of Animal Sciences, Purdue University, West Lafayette, Indiana, United States of America; 2 University of Sao Paulo/ESALQ, Piracicaba, SP, Brazil; 3 Food Animal Production Medicine, School of Veterinary Medicine, Purdue University, Indiana, United States of America; 4 Department of Agronomy, Purdue University, West Lafayette, Indiana, United States of America; U. S. Salinity Lab, United States of America

## Abstract

Obesity leads to changes in the gut microbial community which contribute to the metabolic dysregulation in obesity. Dietary fat and fiber affect the caloric density of foods. The impact of dietary fat content and fiber type on the microbial community in the hind gut is unknown. Effect of dietary fat level and fiber type on hindgut microbiota and volatile fatty acid (VFA) profiles was investigated. Expression of metabolic marker genes in the gut, adipose tissue and liver was determined. A 2×2 experiment was conducted in pigs fed at two dietary fat levels (5% or 17.5% swine grease) and two fiber types (4% inulin, fermentable fructo-oligosaccharide or 4% solka floc, non-fermentable cellulose). High fat diets (HFD) resulted in a higher (P<0.05) total body weight gain, feed efficiency and back fat accumulation than the low fat diet. Feeding of inulin, but not solka floc, attenuated (P<0.05) the HFD-induced higher body weight gain and fat mass accumulation. Inulin feeding tended to lead to higher total VFA production in the cecum and resulted in a higher (P<0.05) expression of acyl coA oxidase (ACO), a marker of peroxisomal β-oxidation. Inulin feeding also resulted in lower expression of sterol regulatory element binding protein 1c (SREBP-1c), a marker of lipid anabolism. Bacteria community structure characterized by DGGE analysis of PCR amplified 16S rRNA gene fragments showed that inulin feeding resulted in greater bacterial population richness than solka floc feeding. Cluster analysis of pairwise Dice similarity comparisons of the DGGE profiles showed grouping by fiber type but not the level of dietary fat. Canonical correspondence analysis (CCA) of PCR- DGGE profiles showed that inulin feeding negatively correlated with back fat thickness. This study suggests a strong interplay between dietary fat level and fiber type in determining susceptibility to obesity.

## Introduction

Recent analysis of the human hindgut microbiome suggests a strong association between microbiome composition and obesity susceptibility [1]–[3]. These studies also suggest that alteration of the gut microbial community could be an approach for obesity prevention and treatment [1]. However, the study of gut microbiota in human subjects is limited by profound individual variation in microbial community composition and sometimes, ethical concerns. Germ-free mice are often used as an animal model, but there are large differences between mice and humans in their physiology and gut microbial communities, primarily due to the significant differences in gut architecture and dietary requirements between the two species. The pig is an ideal animal for investigating the effect of dietary components on bacterial communities and metabolic changes because of similarities in its dietary requirements, and the anatomy and physiology of its digestive tract with that of humans [2].

Dietary fiber, made of carbohydrates and lignin, are resistant to degradation in the upper gut [4]. Fiber has multiple effects on the body, such as, regulation of host gut bacterial community and hind gut fermentation and health [5], [6]. Fiber is thought to alleviate weight gain partly due to its effect in reducing energy density of the diet and increasing satiety [7]. Soluble fiber slows macronutrient absorption and gastric emptying and reduces total and low-density lipoprotein (LPL) concentrations [8]. Likewise, insoluble fiber from cereal and whole grains improve insulin sensitivity and reduces the risk of type 2 diabetes [9]. Therefore, there is a preponderance of evidence on the beneficial effect of dietary fiber on health and metabolic status in humans and animals. However, the extent to which dietary fiber regulates metabolism beyond the gastrointestinal tract is still unclear.

Gut microbes play a key role in the regulation of energy metabolism and fat storage [10]. Although cellulose and inulin are both dietary fiber types, inulin is more highly fermentable than cellulose in the hindgut by humans and pigs and thus could differentially impact the gut microbiome and whole body metabolism. The extent to which the two fiber types alter the gut microbiome in the pig and the interaction of dietary fat content on the fiber types is currently unknown. Therefore, we have used the pig model to investigate the impact of dietary fat content on gut microbial community structure characterized by denaturing gradient gel electrophoresis (DGGE) of 16S rRNA gene PCR products [11] and metabolic markers by fiber types that differ in the degree of fermentability.

## Materials and Methods

### Animals and Experimental Diets

This study was carried out in strict accordance with the recommendations in the Guide for the Care and Use of Laboratory Animals of the National Institutes of Health. The Purdue Animal Care and use Committee (PACUC) approved the protocol. Animals were held under a controlled environment at the Purdue Animal Sciences Research and Extension Center swine facility and all efforts were made to minimize discomfort. Thirty two female pigs (3 months old, initial BW = 10.2±0.15 kg) were randomly allocated to 4 treatments in a 2×2 factorial arrangement with two dietary fat levels (5% or 17.5% swine grease) and two types of fiber (4% inulin or solka floc). Thus we have four treatments: 1) high fat diet with solka floc (HFD Sol), 2) low fat diet with solka floc (LFD Sol), 3) high fat diet with inulin (HFD Inu), and 4) low fat diet with inulin (LFD Inu). Nutrient composition of experimental diets is presented in Table S1. There were 8 pigs per treatment. To minimize differences in intestinal physiology due to gut fill, pigs on the HFDs were pair fed with those on the LFDs for 12 weeks. Feed intake and body weights were recorded weekly. Pigs were humanely sacrificed at the end of the experimental period using approved animal care procedures. Blood and tissue samples (subcutaneous and mesenteric adipose tissue, duodenum, jejunum, ileum and liver) were collected for analysis. Cecal samples were collected and frozen at −20°C until analyzed.

### Volatile Fatty Acid Analysis

Volatile fatty acid (VFA) determination was conducted on metaphosphoric acid derived samples using gas chromatographic methods described by Playne [12]. Hewlett Packard HP 6890 gas chromatography equipment was used for determination of propionate, acetate, butyrate, valerate, isobutyrate, and isovalerate concentrations.

### Cecal Bacteria Determination by PCR-DGGE

Total bacterial DNA was extracted from ceca samples using the FastDNA® SPIN Kit for Soil (MP Biomedicals, Irvine, CA) per manufacturer’s instructions with the exception that 100 mg of ceca samples was used instead of soil [13]. Samples were completely thawed overnight at 4°C and homogenized before being used for DNA extraction. Extracted DNA was quantified by fluorometric analysis on a NanoDrop ND-3300 instrument (Thermo Fisher Scientific Inc., Waltham, MA) using calf thymus DNA as a standard. Microbial community structure was determined using PCR-DGGE [11]. The V3 region of bacterial 16S rRNA gene was amplified by PCR using primers PRBA338F and PRUN518R (Table S2). The PCR reaction mixture contained: 1× PCR buffer (New England BioLabs, Inc., Ipswich, MA), 0.8 mM dNTPs, 0.05% bovine serum albumin (BSA), 0.5 µM of each primer, 2.5 U Taq polymerase (New England BioLabs, Inc., Ipswich, MA), and 1 ng of DNA template in a 50 µL reaction volume. Reactions were carried out on a PTC-100 thermocycler (MJ Research Inc., Watertown, MA) using the following program: initial denaturation at 94°C for 5 min, followed by 30 cycles of denaturation at 92°C for 30 s, annealing at 55°C for 30 s, and extension at 72°C for 30 s, and a final extension step at 72°C for 15 min. The quality and quantity of PCR amplicons were initially determined by agarose gel electrophoresis, then equal amounts of PCR products were separated on DGGE gels composed of 8% (wt/vol) polyacrylamide gels (acrylamide : bisacrylamide = 37.5∶1) in 1× TAE buffer (40 mM Tris, 20 mM Acetate, 1.0 mM EDTA) using the DCode™ Universal Mutation Detection System (Bio-Rad Laboratories, Hercules, CA). DGGE was performed at 60°C at 200V for 4 h and then for 3 h at 100 V, using denaturing gradient ranges from 30 to 75%, 40 to 60%, and 40 to 57.5% (100% denaturant contained 7 M urea and 40% [vol/vol] deionized formamide). Standards were included on all gels to facilitate comparison between gels. After electrophoresis, gels were stained with SYBR® Green I (BioProducts, Rockland, ME, USA) and then digitized on a UV transilluminator (UVP BioImaging system, UVP LLC, Upland, CA) [14].

### Analysis of 16S rRNA Gene PCR-DGGE Fingerprints

PCR-DGGE fingerprint profiles were analyzed using BioNumerics software (Version 2.0; Applied Maths, Kortrijk, Belgium). The presence and absence of bands in profiles were made into a binary matrix for quantitative comparison between different treatments. The number of bands was counted to estimate richness of the most abundant bacterial populations in each sample [15]. Similarities between the banding patterns were analyzed using Dice pairwise coefficients [16]. Hierarchical clustering of the Dice similarity matrix was determined using unweighted pair group method with arithmetic averages (UPGMA) [17]. Principal component analysis (PCA), a multivariate analysis method, was used to calculate the contribution of each variable and generate a 3-dimensional rendering of sample clustering according to their degree of similarity [18].

### Single Band Analysis and Nucleotide Sequence Determination

Based on binary data of the presence or absence of each band in the two fiber types, Fisher’s exact test (SAS version 9.0, SAS Inst. Inc., Cary, NC) was used to analyze the effect of fiber type on each individual band/population. Based on the Fisher’s exact test, the bands that were most significantly affected by fiber type were excised from the DGGE gel, eluted in sterilized PCR water and re-amplified with primers PRBA338F and PRUN518R without the GC clamp. PCR amplicons from bands migrating the same distance extracted from four different inulin-fed pigs were cloned into pGEM-T Easy cloning vector (Promega, Madison, WI) and sequenced in both forward and reverse directions at the Purdue Genomics Center. Nucleotide sequence was compared to the National Center for Biotechnology Information (NCBI) using BLASTn [19] and SeqMatch in the Ribosomal Database Project (RDP) [20] database. The sequences have been deposited into the EMBL database, accession numbers HF584754–HF584759.

### Cecal Bifidobacterium and Bacteroides Abundance Analysis by Real-time Quantitative PCR

Quantitation of *Bifidobacterium*, *Bacteroides* and total Bacteria abundances in the extracted DNA was performed using the MyiQ real-time PCR detection system (Bio-Rad, Hercules, CA) with the SYBR green RT-PCR mix (SABiosciences, Frederic, MD). Bacteria copy numbers were generated from a standard curve prepared from purified plasmid clones of the16S rRNA gene from *Bifidobacterium bifidum* and *Bacteroides vulgatus*. Gene copy number was calculated from the concentration of the extracted plasmid DNA clone assuming 1.096×10^−12^ g/bp. To eliminate potential differential cell lysis effects on DNA extraction, percentages of *Bifidobacterium* and *Bacteroides* with respect to total Bacteria were then calculated. Primer sequences for *Bifidobacterium*, *Bacteroides* and total Bacteria used for quantitative PCR are presented in Table S2.

### Real-time PCR Analysis of Fatty Acid Oxidation, Fatty Acid Synthesis and Inflammatory Gene mRNA Expression

Liver, adipose tissue and intestinal tissues were removed and snap-frozen in liquid nitrogen and then stored at −80°C. Total RNA was extracted using QIAzol lysis reagent (QIAGEN, USA). RNA extracts were dissolved in nuclease-free water (Ambion, Austin, TX) and quantified using a Nanodrop 1000 instrument (Thermo Scientific, Waltham, MA). Reverse transcription was performed using M-MLV reverse transcriptase (Promega, Madison, WI) with 1 µg RNA. Markers of mitochondrial β-oxidation, peroxisome proliferator-activated receptor gamma coactivator 1-alpha (PGC1α) and carnitine palmitoyl transferase 1-alpha (CPT1α) and markers of peroxisomal β-oxidation, acyl-CoA oxidase I (ACO) and peroxisome proliferators activate receptor alpha (PPARα), were analyzed. Markers of lipid anabolism, SREBP-1c and (fatty acid synthase) FAS, were also analyzed. Interleukin-6 (IL-6) and tumor necrosis factor α (TNFα) were selected as inflammatory markers. The primers used for reverse transcriptase qPCR are listed in Table S3. Quantitation of mRNA expression was performed using the MyiQ real-time PCR detection system (Bio-Rad, Hercules, CA) with the SYBR green RT-PCR mix (SABiosciences, Frederic, MD). Abundance of mRNA transcripts was normalized to 18S rRNA (liver and adipose tissue) or GAPDH (intestinal tissue) using Pfaffl’s method [21].

### Statistical Analysis

In order to compare the changes in bacterial community structure that came from different fat levels and fiber types, two-way analyses of similarity (ANOSIM) [22] was applied to the band binary matrix from DGGE fingerprints analysis. A two-way non-parametric permutation analysis of variance (perMANOVA) was also applied to evaluate the bacterial differences under different diet treatments. Both ANOSIM and perMANOVA were determined using PAST (PAleontological Statistics) v2.15 software [23]. The response of single bands in the populations to different fiber diets was determined using Fisher’s exact test in SAS version 9.0 (SAS Inst. Inc., Cary, NC). The p-value from Fisher’s exact test was corrected by the Holm-Bonferroni method.

The binary matrices of PCR-DGGE bacterial profiles were further analyzed by canonical correspondence analysis (CCA) to determine the possible experimental variables that were correspondent to gut bacterial community structure. CCA is a multivariate method to illustrate the relationships between biological structures of species and environmental variables, which is widely used in community ecology [24]. Canonical correspondence analysis was performed using PC-ORD software version 6.07 [25]. The scaling options in PC-ORD software used were to optimize samples and perform biplot scaling. The Monte Carlo test method was used to test variance correspondence to PCR-DGGE data with the number of randomized permutations at n = 999 and at the significance level of α<0.05.

Our analysis combined the performance data with bacteria fingerprints to find potential connections between performance variables and bacterial community structure.

Statistical analysis of performance data and gene expression data was conducted using the GLM program in SAS version 9.0 (SAS Inst. Inc., Cary, NC). The Tukey multiple comparison test was used to determine significant mean differences. Data was expressed as mean ± SE. P values less than 0.05 were considered statistically significant whereas P values between 0.05 and 0.1 were considered as showing a trend.

## Results

### Effect of Fiber Type and Fat Content on Pig Growth Performance

Pigs fed HFD had a higher final body weight, average daily weight gain, feed conversion ratio, total back fat (TBF) thickness and inner back fat (IBF) thickness (Table 1) than those fed the LFD. However, fiber type did not significantly influence body weight gain, and feed conversion but pigs fed inulin diets tended (P = 0.09) to have thinner inner back fat than those fed solka floc. There was a trend for fiber × fat level interaction (P = 0.09) such that the HFD-induced body weight and total back fat thickness were significantly suppressed when animals were fed inulin compared to those on HFD fed solka floc (Table 1). There was no effect of fiber type or fat level on serum glucose concentration (Table 1).

**Table 1:** Effect of dietary fiber type and fat content on pig growth performance.

	LFD		HFD			Fat level		Fiber		P-value		
Growth Performance	Inu	Sol	Inu	Sol	SE	LFD	HFD	Inu	Sol	Fat	Fiber	Fat*Fiber
Body Weight (kg)	63.59^ab^	61.75^b^	67.53^ab^	71.40^a^	3.38	62.67	69.47	66.57	65.56	0.057	0.77	0.41
Weight Gain (g/d)	681.20^bc^	642.32^c^	812.42^ab^	851.03^ab^	38.14	661.76^b^	831.72^a^	746.81	746.67	0.01	0.99	0.34
Feed conversion ratio (g/kg)	402.93^bc^	378.67^c^	477.72^ab^	493.77^a^	20.78	390.80^b^	485.74^a^	440.32	436.34	0.01	0.85	0.36
Back Fat (Total cm)	2.70^b^	2.59^b^	3.11^ab^	3.49^a^	0.16	2.64^b^	3.30^a^	2.91	3.04	0.01	0.43	0.15
Back Fat (Inner cm)	1.56^c^	1.56^c^	1.89^b^	2.25^a^	0.10	1.56^b^	2.07^a^	1.73	1.91	0.01	0.09	0.09
Blood Glucose (mg/dL)	90.38	81.00	73.13	63.33	10.38	85.69	68.23	81.75	72.17	0.12	0.38	0.98

LFD, low fat diet-fed pigs; HFD, high fat diet-fed pigs; Inu, inulin diet-fed pigs; Sol, solka floc diet fed pigs. Data are presented as least-square means ± SE. Different letters within rows indicate significant differences (P<0.05).

### Effects of Fiber Type and Fat Content on Concentrations of Volatile Fatty Acids in the Cecum

HFD fed pigs had a lower concentration of acetate, propionate and butyrate than the LFD fed pigs (Table 2). Inulin feeding tended (P = 0.07) to yield a higher concentration of propionate without a significant effect on the concentration of acetate and butyrate, compared with solka floc. Inulin also appeared to eliminate the effect of HFD on VFA concentration such that animals on HFD but on inulin had higher VFA concentrations than those on HFD, but fed solka floc (Table 2).

**Table 2:** Effect of dietary fiber type and fat content on concentration of volatile fatty acids in the cecum.

	LFD		HFD			Fat level		Fiber		P-value		
VFAs (µM)	Inu	Sol	Inu	Sol	SE	LFD	HFD	Inu	Sol	Fat	Fiber	Fat*Fiber
Acetate	45.90^ab^	51.52^a^	44.47^ab^	37.95^b^	2.87	48.71^a^	41.25^b^	45.19	44.74	0.02	0.88	0.05
Propionate	26.87^a^	23.15^ab^	21.98^ab^	15.57^b^	2.58	25.01^a^	18.78^b^	24.43^a^	19.36^b^	0.03	0.07	0.62
Butyrate	17.73^a^	17.44^a^	15.17^a^	11.56^b^	1.75	17.59^a^	13.37^b^	16.45	14.50	0.03	0.31	0.39
Total	90.51^a^	92.56^a^	81.63^ab^	65.09^b^	6.17	91.54	73.36	86.07	78.83	0.01	0.29	0.18

LFD, low fat diet-fed pigs; HFD, high fat diet-fed pigs; Inu, inulin diet-fed pigs; Sol, solka floc diet fed pigs. Data are presented as least-squares means ± SE. Different letters within rows indicate significant differences (P<0.05).

### Effect of Fiber Type and Fat Content on Cecal Microbial Community Structure

**Figure 1:**
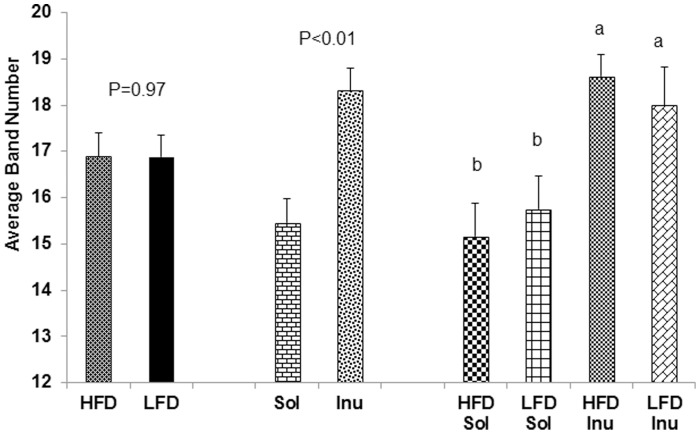
Band numbers from the PCR-DGGE profiles of cecal microbial communities. Dietary fiber type and fat content affected band numbers of 16S rRNA gene PCR-DGGE profiles of cecal microbial communities. LFD, low fat diet-fed pigs; HFD, high fat diet-fed pigs; Inu, inulin diet-fed pigs; Sol, solka floc diet fed pigs. Bars represent least-squares means ± SE. Different letters on bars indicate significant difference (P<0.05).

Each band in the PCR-DGGE profile theoretically represented one species/population of bacteria. The number of the different bands provides an estimation of the number of abundant populations in each sample [15]. Feeding inulin resulted in significantly higher number of visible bands compared with solka floc (P<0.01). There was no observable effect of dietary fat level on visible band numbers (Fig. 1).

**Figure 2:**
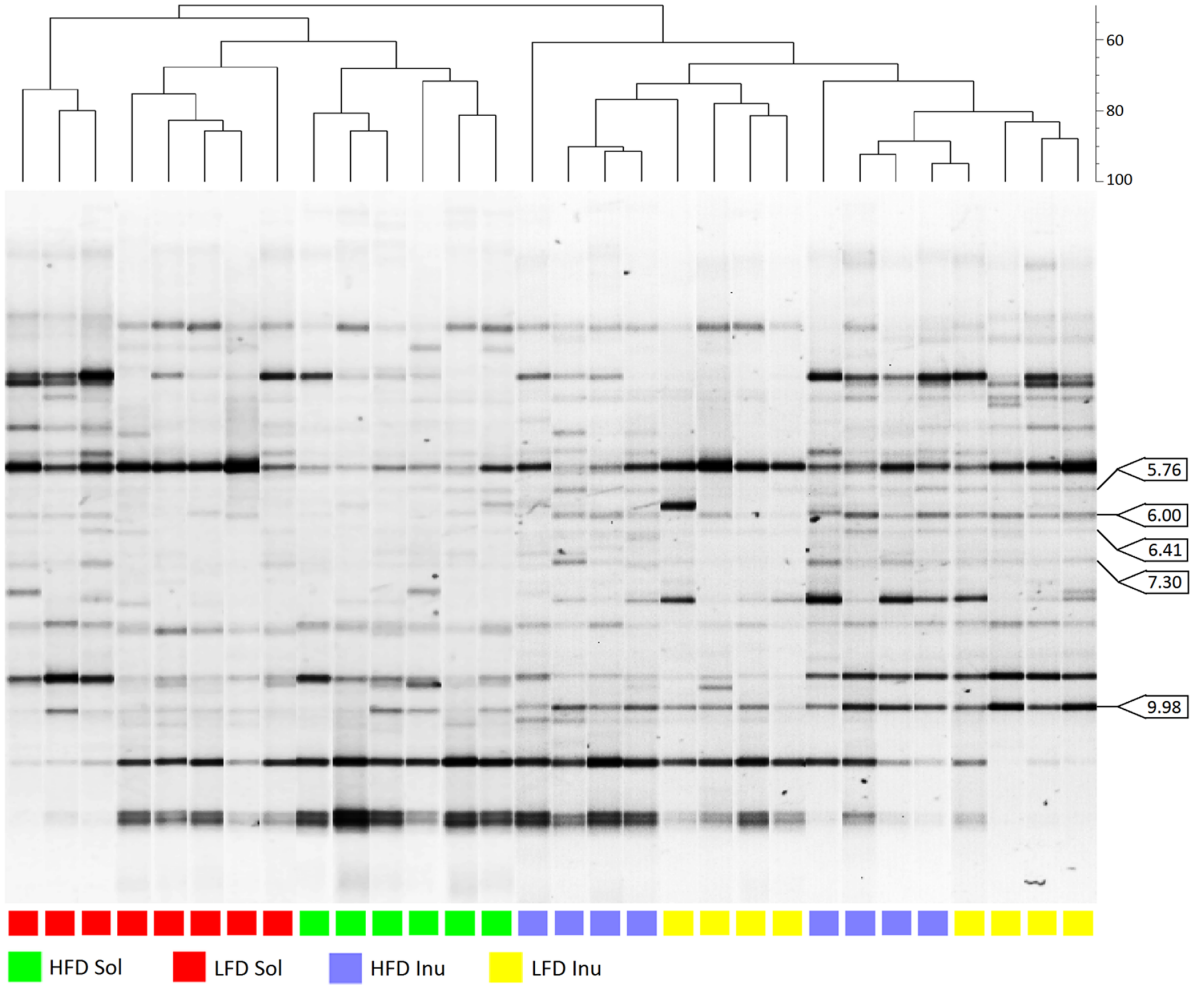
Dendrogram of PCR-DGGE profiles of pig cecal bacterial community. Dendrogram of PCR-DGGE profiles of pig cecal bacterial community on diets with different fat levels and fiber types. The unweighted pair group method with arithmetic mean (UPGMA) dendrogram was generated based on a matrix of pairwise Dice similarity comparisons of 16S rRNA gene PCR-DGGE fingerprints. LFD, low fat diet-fed pigs; HFD, high fat diet-fed pigs; Inu, inulin diet-fed pigs; Sol, solka floc diet fed pigs. The scaled bar on the upper-left indicates percentage similarity coefficients. Labels indicate bands that are significantly associated with the inulin diet.

The UPGMA dendrogram of Dice pairwise similarity indices of PCR-DGGE profiles illustrated separation of the profiles into two distinct clusters that corresponded with the two different fiber types fed to the pigs (Fig. 2). The solka floc cluster was further divided into three sub-clusters, which were separated partly by fat level with one cluster associating with the HFD, and the other two clusters associating with the LFD. The inulin cluster separated into two sub-clusters but these two sub-clusters did not correspond to dietary fat level. These results indicate that inulin had the predominant effect on microbial community structure and dietary fat level had a lesser influence.

**Figure 3:**
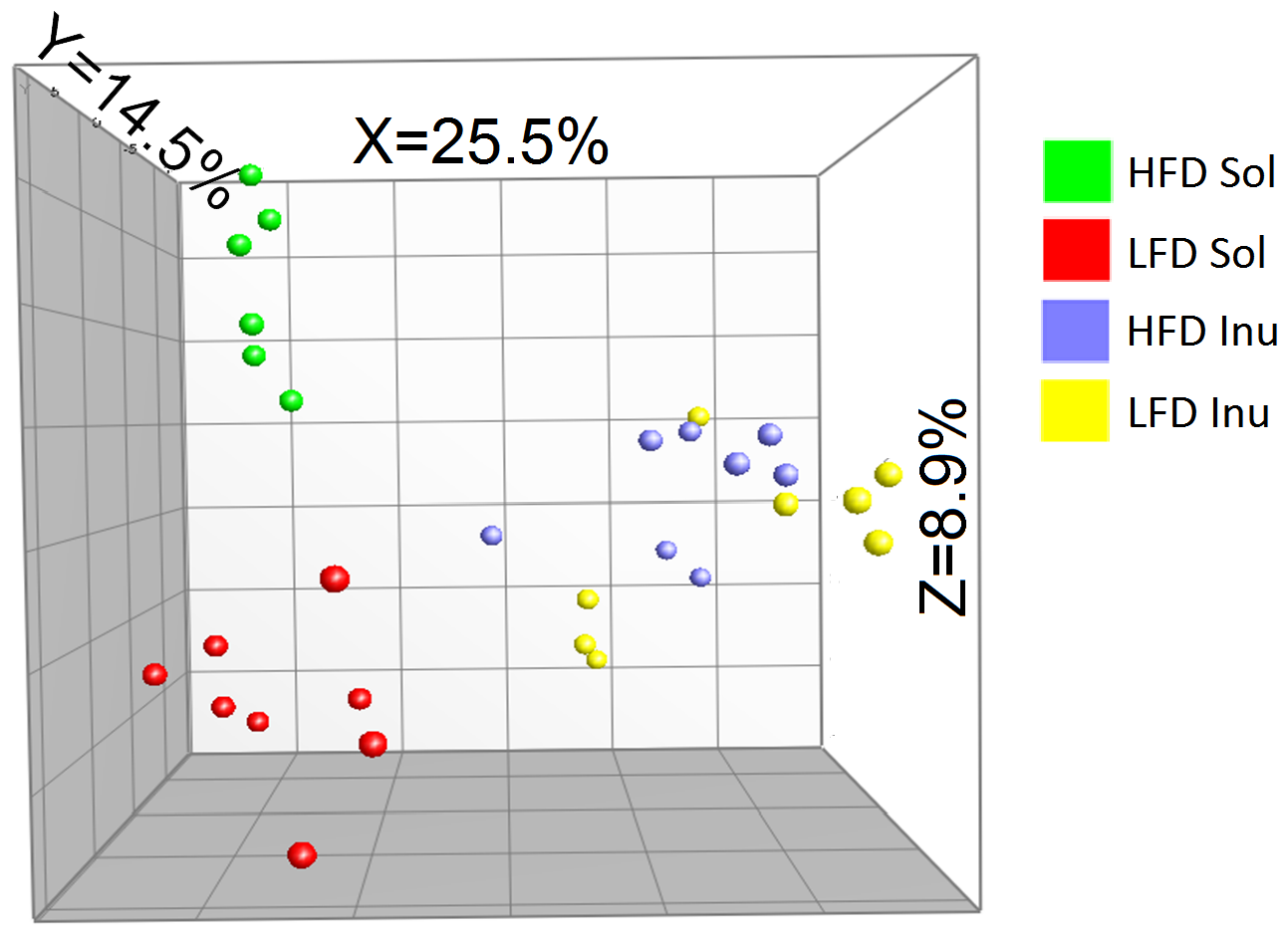
Principal component analysis of pig cecal bacterial community. Principal component analysis of pig cecal bacterial community was based on comparisons of16S rRNA gene PCR-DGGE fingerprint profiles. LFD, low fat diet-fed pigs; HFD, high fat diet-fed pigs; Inu, inulin diet-fed pigs; Sol, solka floc diet fed pigs. Values on the axis indicate variation percentages contributed to the first three principal components (X, Y, Z, respectively).

Principal component analysis (PCA) of the PCR-DGGE fingerprint profiles (Fig. 3) also indicated that samples associated in distinct groups similar to the results of the cluster analysis of the PCR-DGGE profiles presented in Fig. 2. The PCA analysis again revealed a more prominent effect of fiber type on the clustering of bacteria species. In solka floc-fed pigs, the different levels of fat also show the prominent effect on the clustering of bacteria species, however, such effect was low in inulin-fed pigs.

Based on band binary matrix from DGGE profile of bacterial communities, significant differences were observed between inulin and solka floc diets (ANOSIM R = 0.8548, P<0.01). Although cluster analysis indicated dietary fat level had a lower influence, especially with inulin diets, the differences in bacterial communities between HFD and LFD were still significant (ANOSIM R = 0.3641, P<0.01). Two-way perMANOVA analysis also produced similar results (fiber P<0.01, fat level P<0.01, fat x fiber interaction, P = 0.014).

**Figure 4:**
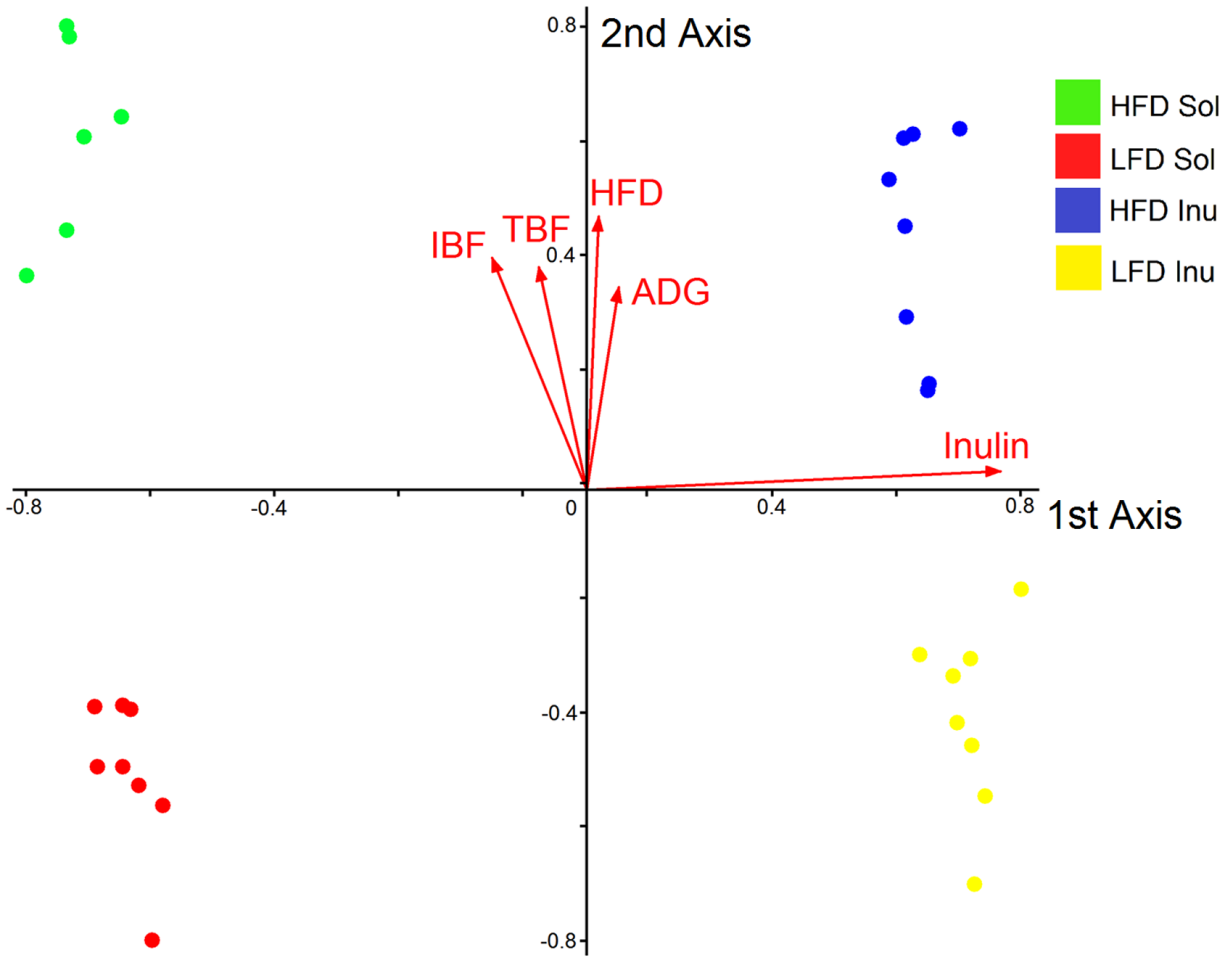
Canonical correspondence analysis (CCA) of bacterial PCR-DGGE community profiles from pig cecal samples. The colored spots represent samples from four different treatments. Each red line represents a variable, IBF: inner back fat, TBF: total back fat and ADG: average daily gain. The variables significantly (P<0.05) correlate to the differences in bacterial community structure. The length of each line represents the contribution of each variable to the bacterial community structure difference.

Based on canonical correspondence analysis (CCA), dietary fiber, fat level, total back fat, inner back fat and ADG were significantly associated with PCR-DGGE profiles of bacteria community structure (Fig. 4). The first two axes explained 15.2 and 7.5 percent of the variance in the CCA. Inulin had the greatest association with bacterial community data along the first axis, and such association was different from those of HFD and other variables whose principle correspondence was with the second axis. High fat diet (HFD) had the strongest effect on the second axis, but in a similar way as average daily gain (ADG), inner and total back fat (Fig. 4). There were also correlations between these variables: ADG, inner and total back fat. Inner and total back fat positively correlated to HFD with correlation coefficients of 0.620, 0.637 and 0.579, respectively. This positive correlation probably accounted for their similar effect on bacteria community and also implied that bacteria community profile may have a relationship to fat accumulation and weight gain. Inulin was negatively correlated to inner and total back fat with correlation coefficients of −0.158 and −0.060, respectively. This negative correlation between inulin and back fat indicates the greater potential capacity for inulin to lower fat accumulation than solka floc.

### Determination of Sequence of Fiber-responsive Bacteria

Fisher’s exact tests indicate that a total number of five bacteria bands were significantly different in response to the two fiber types (Table 3). These five bands (marked in Fig. 2) were present in almost all the inulin-fed pigs and absent in all solka floc-fed pigs. Among these bands unique to inulin, bands 9.98 and 6.00, which were the most intense two bands on the PCR-DGGE gel, were excised from individual pigs and multiple clones from each band were sequenced. For band 9.98, multiple sequence results were 100% identical and the sequence matched with 97% identity to *Catenibacterium mitsuokai* JCM 10609^T^ (AB030224), in the phylum *Firmicutes* and family *Erysipelotrichaceae*. For band 6.00, multiple sequence results were 100% identical from two pigs and the sequence matched with 100% identity to *Blautia wexlerae* WAL 14507^T^ (EF036467), in the phylum *Firmicutes* and family *Lachnospiraceae*. Due to the short sequence of these bands only the genus designation is reliable at this point. Sequences for bands 5.76, 6.41 and 7.30 could not be obtained likely because they were relatively weak and in close proximity to other bands.

**Table 3:** Fisher’s exact test results on bands/bacteria associated with different fiber types.

Band name	Responded fiber^1^	Sol-BP^2^	Sol-BA^2^	Inu-BP^2^	Inu-BA^2^	Fisher’s exact test^3^
9.98	Inu only	0	14	16	0	0.001
6.00	Inu only	0	14	14	2	0.001
7.30	Inu only	0	14	12	4	0.001
6.41	Inu predominant	3	11	15	1	0.005
5.76	Inu predominant	3	11	14	2	0.032

1 Inu only, bands present only in inulin diet-fed pigs. Inu predominant, bands present in both inulin and solka floc fed pigs, but predominant in inulin diet with (P<0.05).

2 Sol-BP, bands present in solka floc fed pigs. Sol-BA, bands absent in solka floc pigs. Inu-BP, bands present in inulin fed pigs. Inu-BA, bands absent in solka floc pigs.

3 P-value of Fisher’s exact test was corrected using the Holm-Bonferroni method.

### Effect of Dietary Fiber Type and Fat Content on Percentages of Bifidobacterium and Bacteroides to Total Bacteria

Based on quantification of 16S rRNA genes of purified *Bifidobacterium bifidum* and *Bacteroides vulgatus*, the average gene copy numbers using Bacteria primers were 2.90×10^6^±1.05×10^5^/ng DNA and 3.62×10^6^±1.56×10^5^/ng DNA, respectively, which had the same order of magnitude as previously reported for pigs [26]. Quantification of 16S rRNA gene copy percentages of *Bifidobacterium* and *Bacteroides* relative to total Bacteria indicated that HFD feeding resulted in a significantly (P<0.01) higher relative percentage of *Bacteroides* but not *Bifidobacteria*, compared with LFD. There was no significant effect of fiber type on these bacteria. However, in HFD fed pigs, inulin resulted in a higher percentage of *Bacteroides* than those on solka floc diets (Table 4).

**Table 4:** Effect of dietary fiber type and fat content on percentages of *Bifidobacterium* and *Bacteroides* to total bacteria in cecal content.

	LFD		HFD			Fat level		Fiber		P-value		
Bacteria	Inu	Sol	Inu	Sol	SE	LFD	HFD	Inu	Sol	Fat	Fiber	Fat*Fiber
*Bifidobacterium* (×10^3^ copy no./ng)	1.33	2.04	1.45	2.00	0.45	1.68	1.72	1.39	2.02	0.92	0.14	0.84
Total Bacteria based on *Bifidobacterium* (×10^6^ copy no./ng)	2.85	3.24	2.37	3.23	0.32	3.04	2.80	2.61	3.24	0.38	0.03	0.40
*Bifidobacterium* (%)	0.046	0.062	0.063	0.066	0.014	0.054	0.064	0.055	0.064	0.42	0.48	0.63
*Bacteroides* (×10^3^ copy no./ng)	0.36^b^	1.66^a^	3.08^a^	1.43^a^	0.65	1.01	2.25	1.72	1.54	0.01	0.06	0.01
Total Bacteria based on *Bacteroides* (×10^6^ copy no./ng)	3.29	4.00	3.17	4.14	0.48	3.64	3.65	3.23	4.07	0.98	0.06	0.77
*Bacteroides* (%)	0.01^b^	0.05^b^	0.11^a^	0.04^b^	0.02	0.03^b^	0.07^a^	0.06	0.04	0.01	0.16	0.01

LFD, low fat diet-fed pigs; HFD, high fat diet-fed pigs; Inu, inulin diet-fed pigs; Sol, solka floc diet fed pigs. Data are presented as least-squares means ± SE. Different letters within rows indicate significant differences (P<0.05).

### Effect of Dietary Fiber Type and Fat Content on Gene Expression in Adipose Tissue, Liver and Intestinal Sections

Compared with solka floc-fed pigs, inulin feeding resulted in higher mRNA expression of Acyl-CoA oxidase (ACO) in the liver (P = 0.02) (Table 5) and subcutaneous adipose tissue (P = 0.03) (Table 6). Expression of CPT1α was also higher in HFD fed pigs compared with LFD in the liver (P<0.01) and subcutaneous fat (P = 0.04). However, fiber type did not affect expression of CPT1α in the liver and subcutaneous fat. In the mesenteric adipose tissue, HFD was associated with a tendency (P = 0.07) for higher CPT1α mRNA expression, but fiber type did not affect CPT1α expression in this depot as well (Table 7).

**Table 5:** Effect of dietary fiber type and fat content on gene expression in the liver.

	LFD		HFD			Fat level		Fiber		P-value		
Gene	Inu	Sol	Inu	Sol	SE	LFD	HFD	Inu	Sol	Fat	Fiber	Fat*Fiber
ACO	1.96^a^	1.25^ab^	1.50^ab^	0.94^b^	0.36	1.61	1.22	1.73^a^	1.10^b^	0.16	0.02	0.56
CPT1α	0.77^b^	0.88^b^	1.59^a^	1.72^a^	0.25	0.83^b^	1.82^a^	1.35	1.30	0.01	0.91	0.89
FAS	0.96	0.87	1.47	1.06	0.19	0.91	1.26	1.21	0.96	0.09	0.23	0.44
SREBP-1c	2.01	1.60	1.11	0.97	0.43	1.81	1.04	1.56	1.29	0.10	0.54	0.76
PGC1α	1.24	1.28	1.17	1.05	0.23	1.26	1.11	1.20	1.16	0.54	0.86	0.75
PPARα	1.05	1.24	1.07	1.13	0.23	1.15	1.10	1.06	1.19	0.41	0.72	0.80
IL-6	1.13	1.12	1.04	1.33	0.36	1.12	1.19	1.08	1.23	0.78	0.55	0.53
TNFα	1.11	1.04	1.39	1.41	0.25	1.07	1.40	1.25	1.23	0.23	0.93	0.75

LFD, low fat diet-fed pigs; HFD, high fat diet-fed pigs; Inu, inulin diet-fed pigs; Sol, solka floc diet fed pigs. Data are presented as least-square means ± SE. Different letters within rows indicate significant differences (P<0.05).

**Table 6:** Effect of dietary fiber type and fat content on gene expression in subcutaneous adipose tissue.

	LFD		HFD			Fat level		Fiber		P-value		
Gene	Inu	Sol	Inu	Sol	SE	LFD	HFD	Inu	Sol	Fat	Fiber	Fat*Fiber
ACO	1.30	0.94	1.29	0.88	0.15	1.12	1.08	1.30^a^	0.91^b^	0.82	0.03	0.88
CPT1α	0.96	0.88	1.36	1.25	0.17	0.92^b^	1.31^a^	1.16	1.07	0.04	0.63	0.93
FAS	0.99	0.91	1.13	1.06	0.10	0.95	1.10	1.06	0.98	0.20	0.52	0.96
SREBP-1c	1.53^a^	1.56^a^	0.67^b^	0.89^ab^	0.19	1.55^a^	0.78^b^	1.10	1.22	0.01	0.56	0.64
PPARα	1.31	1.21	0.96	0.81	0.17	1.26	0.88	1.14	1.01	0.06	0.52	0.88
IL-6	0.84	0.92	2.02	2.48	0.51	0.88^b^	2.25^a^	1.43	1.70	0.02	0.71	0.89
TNFα	0.87	0.96	1.06	1.26	0.14	0.92	1.16	0.96	1.11	0.12	0.33	0.73

LFD, low fat diet-fed pigs; HFD, high fat diet-fed pigs; Inu, inulin diet-fed pigs; Sol, solka floc diet fed pigs. Data are presented as least-square means ± SE. Different letters within rows indicate significant differences (P<0.05).

**Table 7:** Effect of dietary fiber type and fat content on gene expression in the mesenteric adipose tissue.

	LFD		HFD			Fat level		Fiber		P-value		
Gene	Inu	Sol	Inu	Sol	SE	LFD	HFD	Inu	Sol	Fat	Fiber	Fat*Fiber
ACO	1.11	1.37	0.83	1.66	0.34	1.24	1.24	1.10	1.39	0.98	0.31	0.06
CPT1α	1.17	0.90	1.39	1.72	0.44	1.03	1.56	1.28	1.59	0.07	0.78	0.25
FAS	0.94	1.00	1.16	1.25	0.13	0.97	1.20	1.05	1.12	0.10	0.59	0.97
SREBP-1c	1.68	3.52	0.96	2.06	0.70	2.60	1.51	1.32^b^	2.79^a^	0.22	0.03	0.65
PGC1α	0.88	0.85	0.76	0.61	0.22	0.87	0.68	0.73	0.82	0.44	0.79	0.88
PPARα	1.45	1.74	0.85	1.47	0.30	1.59	1.16	1.15	1.60	0.18	0.16	0.62
IL-6	0.93	1.28	1.36	1.29	0.37	1.11	1.32	1.14	1.29	0.60	0.73	0.62
TNFα	1.47	0.69	2.33	1.13	0.51	1.08	1.73	1.90	0.91	0.25	0.09	0.70

LFD, low fat diet-fed pigs; HFD, high fat diet-fed pigs; Inu, inulin diet-fed pigs; Sol, solka floc diet fed pigs. Data are presented as least-square means ± SE. Different letters within rows indicate significant differences (P<0.05).

For lipid anabolism related genes, LFD feeding resulted in higher expression of SREBP-1c in subcutaneous fat (P<0.01) and a trend (P = 0.10) for a higher expression in the liver as well. Compared with solka floc-fed pigs, expression of SREBP-1c was lower in inulin fed pigs (P = 0.03) in the mesenteric fat. Fat level and fiber type did not significantly affect the expression of FAS.

In HFD fed pigs, there was a tendency (P = 0.09) for a higher TNFα mRNA expression in the mesenteric fat, and significantly higher (P = 0.02) IL-6 expression in the subcutaneous fat.

There was no significant effect of fiber type and fat level on the expression of genes in the intestinal sections (Table S4). In the cecum, solka floc feeding tended (P = 0.07) to result in higher IL-6 expression compared to inulin. However, this tendency was reversed (P<0.03) in the jejunum. Feeding solka floc also tended (P<0.06) to result in lower TNFα expression in the jejunum.

## Discussion

Chronic intake of high fat diet is associated with being overweight and obese [27]. However, the effect of dietary fiber on obesity susceptibility and long-term body weight gain is still controversial. Although both soluble and insoluble fiber consumption has been found to be inversely associated with body weight gain [28], [29], long term study of fiber consumption indicate soluble fiber could increase fermentation and decrease net energy loss in the fecal matter, leading to increased body weight and obesity [30]. In our study, two types of fiber that differed in their fermentability were tested. Solka floc, a cellulosic material is less fermentable than inulin, which is mainly composed of fructo-oligosaccharides. Although we observed increased concentrations of volatile fatty acids in the inulin-fed pigs, the extra energy from the VFAs was not high enough to lead to a significant increase in body weight gain, compared with solka floc-fed pigs. Instead, feeding inulin significantly reduced HFD-induced body weight gain and body fat accumulation. Several mechanisms may explain the reduction in weight gain attributable to inulin. Evidence suggests that insoluble fiber induces PGC1α expression, a transcription factor that regulates expression of genes involved in mitochondrial fatty acid oxidation, thus increasing mitochondrial β-oxidation and reduction in fat mass [30]. However, since we did not observe any significant difference in the PGC1a expression between inulin and solka floc based diets, alteration of mitochondrial β-oxidation may not contribute significantly to the different responses between inulin and solka floc to HFD. Inulin feeding led to increased expression of ACO, especially in the liver and subcutaneous fat. This observation is consistent with the potential effect of inulin in stimulating peroxisomal β-oxidation, another key pathway for fatty acid oxidation [31]. Although our experiment was not set up to measure fatty acid oxidation, inulin could preferentially stimulate peroxisomal, rather than mitochondrial, fatty acid oxidation. Mechanisms by which inulin could stimulate fatty acid oxidation are still unclear, but could involve the increase in the production of short chain fatty acids, which then act on the liver and subcutaneous fat to increase fatty acid oxidation. Unlike the results on effect of fiber type, increasing dietary fat level resulted in increased expression of carnitine palmitoyltransferase-1 (CPT1), a rate limiting enzyme in mitochondrial fatty acid oxidation, in the liver and adipose tissue. This is similar to the reported induction of CPT1 by HFD in rodents [32]. However, any interaction between dietary fat level and fiber type on the expression of both ACO and CPT1α expression cannot be conclusively established at this time. SREBP-1c is an important transcription factor for lipogenesis, regulating the expression of lipogenic genes [33], [34]. Inulin feeding led to a lower expression of SREBP-1c, implying that inulin had the potential to decrease lipid de novo lipogenesis. The expression of SREBP-1c is regulated by liver X receptor (LXR) activation [34], and there is evidence that free fatty acids can suppress its expression by antagonizing activation of LXR [35], [36]. Consistent with this possibility, we also observed reduced expression of SREBP-1c as a result of HFD feeding. In addition, the reduced expression of SREBP-1c in inulin-fed pigs suggests that inulin could antagonize the activation of LXR either through the increased production of short chain fatty acids or yet unknown mechanisms. Taken together, the increased expression of oxidative genes such as ACO and the decreased expression of lipogenic genes such as SREBP-1c could account for the lower fat accumulation in inulin fed pigs.

Gut microbial community plays an important role in host health, especially in the modulation of adiposity [37]. Differences in gut microbial community may partly be contributing to the observed difference in the fat thickness between the fiber types. The community PCR-DGGE profiles separated into two major clusters based on dietary fiber. The inulin diet has a higher number of bands in the DGGE profiles suggesting that a greater number of bacteria populations are supported by inulin and/or its metabolites than solka floc (Table 4). It is also interesting to note that inulin, unlike solka floc, appears to limit the HFD-induced lowering of gut microbial population richness. Similar increases in gut microbial richness were found with feeding chicory root, a major source of inulin, to pigs [5]. Given that back fat thickness and fiber supplementation was significantly associated with bacteria community structure, and inulin feeding negatively correlated to back fat thickness, the potential connection between inulin feeding and gut bacteria communities and reduced back fat thickness can be established. We hypothesize that inulin substantially changes gut bacteria community composition, and consequently alters metabolites types and concentrations, leading to a significant negative impact on fat accumulation.

*Bifidobacterium* and *Bacteroides* are the two microbial genera that have been reported to have significant effect on substrate fermentation in the hind gut and energy homeostasis [38]. In this study, the percentage of cecal *Bacteroides* was relatively higher in inulin-fed pigs with HFD than other treatment, this may account for inulin-suppressing HFD induced fat accumulation. But the relatively low abundance of *Bacteroides* to total bacteria in this study suggests that the potential contribution of this group to the observed weight gain and adiposity is very small. Alternatively, this low abundance of *Bacteroides* may reflect a difference in microbial structure between humans and swine. In swine, *Bacteroides* may constitute a small fraction of the phylum *Bacteroidetes* community and other members of this phylum may be related to obesity. *Bifidobacterium* is another bacterial group that has been shown to have probiotic properties in mammals [39]. *Bifidobacteria* is known to degrade inulin to generate fructo-oligosaccharides [40] and ingestion of inulin stimulates *Bifidobacterium* abundance *in vivo* [41]. However, in this study, feeding inulin did not result in higher levels of *Bifidobacterium,* suggesting that other bacteria may also contribute significantly to the observed animal performance. A metagenomic analysis may be needed in the future to determine the identity of bacteria species that mediate the inulin effect.

However, inulin feeding resulted in some unique bands on the PCR-DGGE profile. Based on our preliminary sequence results, bacteria in the genus *Catenibacterium* and *Blautia* were exclusively present only in the inulin-fed pigs. *Catenibacterium* is Gram-positive and an obligatory anaerobe that utilizes glucose to produce acetic, lactic, butyric and iso-butyric acids [42]. *Blautia* is also Gram-positive and anaerobic. It can utilize many kinds of carbohydrates to produce acetic, lactic acids and ethanol [43]. The association between *Catenibacterium* and *Blautia* with inulin fermentation is not well characterized; however, both of these bacteria belong to the phylum *Firmicutes*. Studies have indicated that bacteria in the phylum *Firmicutes* are enriched by inulin both *in vivo* [3] and *in vitro* [44], [45]. Human and rodent studies also reveal a higher enrichment of *Firmicutes* versus *Bacteroidetes* in the microflora of obese vs. lean subjects [46], [47], although other studies failed to show any association [48]. Our study also established the connection between *Firmicutes* and inulin presence in the diet. Most butyrate-producing bacteria belong to the phylum *Firmicutes* [49]. Therefore, enrichment of *Catenibacterium* in the cecum may have contributed to the increased VFA production with inulin consumption observed in the study. Inulin feeding resulted in increased cecal VFA concentration (propionate and butyrate) (Table 2) and the detected presence of *Catenibacterium* and *Blautia* in the cecal microbiota suggests a possible association between these microbes and VFA production in the porcine hind gut.

Although fiber consumption is linked to reduction in weight gain and fat accumulation [9], [30], there is evidence that fiber consumption could also promote obesity by increasing hind gut energy recovery [30]. Many earlier studies employed a fairly high level of fiber (>10% of diet), and the observed fiber effect in these studies could have been partly due to the dilution of food calories by the high level of fiber. We have employed a lower level of fiber supplementation in this study to limit the fermentative energy derived from inulin while deriving the potential probiotic and anti-inflammatory benefits of fermentable fiber [49]–[51]. Taken together, our results show that feeding inulin significantly limits the effects of HFD on the microbiota and results in a higher hindgut microbial diversity which may regulate the level of metabolites that increase fatty acid oxidation and suppress fatty acid synthesis.

## Supporting Information

Table S1 The nutrient composition of the experimental diets.
(DOCX)

Table S2 PCR primers for amplification of 16S rRNA gene of Bacteria, *Bifidobacterium* and *Bacteroides*.
(DOCX)

Table S3 Primers for fat oxidation and inflammatory genes for Real-time PCR.
(DOCX)

Table S4 Effect of different fiber types and fat content on gene expression in intestinal sections.
(DOCX)
