# Supplementary material for: Dietary Fat Content and Fiber Type Modulate Hind Gut Microbial Community and Metabolic Markers in the Pig
Source: PLoS One. 2013 Apr 3;8(4):e59581. doi: 10.1371/journal.pone.0059581 (PMC3616062; doi:10.1371/journal.pone.0059581)
Supplement: Table S1 — The nutrient composition of the experimental diets. (DOCX) [file pone.0059581.s001.docx]

**Table 1S**. The nutrient composition of the experimental diets

| **Ingredient (% in diet)** | **High fat Solka floc** | **Low fat Solka floc** | **High fat Inulin** | **Low fat Inulin** |
| --- | --- | --- | --- | --- |
| Corn NRC 1998 | 49.235 | 63.735 | 49.235 | 63.735 |
| SBM | 22.640 | 22.640 | 22.640 | 22.640 |
| Swine Grease | 17.500 | 3.000 | 17.500 | 3.000 |
| Solka Floc | 4.000 | 4.000 | 0.000 | 0.000 |
| Inulin | 0.000 | 0.000 | 4.000 | 4.000 |
| DDGS Ren low Lys & fat | 2.500 | 2.500 | 2.500 | 2.500 |
| Limestone | 1.350 | 1.350 | 1.350 | 1.350 |
| MonoCal | 0.740 | 0.740 | 0.740 | 0.740 |
| Swine Vit. Prx | 0.250 | 0.250 | 0.250 | 0.250 |
| TM Prx | 0.125 | 0.125 | 0.125 | 0.125 |
| Se 600 Prx | 0.050 | 0.050 | 0.050 | 0.050 |
| Phytase | 0.100 | 0.100 | 0.100 | 0.100 |
| Salt | 0.350 | 0.350 | 0.350 | 0.350 |
| Lysine-HCL | 0.400 | 0.400 | 0.400 | 0.400 |
| DL-Methionine | 0.120 | 0.120 | 0.120 | 0.120 |
| L-Threonine | 0.160 | 0.160 | 0.160 | 0.160 |
| L-Tryptophan | 0.030 | 0.030 | 0.030 | 0.030 |
| Banmith dewormer | 0.100 | 0.100 | 0.100 | 0.100 |
| Rabon Larvacide | 0.025 | 0.025 | 0.025 | 0.025 |
| Carbadox (10g/lb) | 0.250 | 0.250 | 0.250 | 0.250 |
| Copper Sulfate | 0.075 | 0.075 | 0.075 | 0.075 |
| Diffusion Plus | 0.000 | 0.000 | 0.000 | 0.000 |
| Total | 100.000 | 100.000 | 100.000 | 100.000 |
