# Supplementary material for: Dietary Fat Content and Fiber Type Modulate Hind Gut Microbial Community and Metabolic Markers in the Pig
Source: PLoS One. 2013 Apr 3;8(4):e59581. doi: 10.1371/journal.pone.0059581 (PMC3616062; doi:10.1371/journal.pone.0059581)
Supplement: Table S2 — PCR primers for amplification of 16S rRNA gene of Bacteria, Bifidobacterium and Bacteroides. (DOCX) [file pone.0059581.s002.docx]

**Table 2S**. PCR primers for amplification of 16S rRNA gene of Bacteria, *Bifidobacterium* and *Bacteroides*.

| **Primer** | **16S rRNA target (base number)^*^** | **Primer sequence (5’ to 3’)** | **Anneal Temp.** | | | **References** |
| --- | --- | --- | --- | --- | --- | --- |
| PRBA338F | Bacteria 16S rRNA V3 region (338-358) | **AC TCC TAC GGG AGG CAG CAG | | 55°C | | [[11](#_ENREF_11)] |
| PRUN518R | Bacteria 16S rRNA V3 region (534-518) | ATT ACC GCG GCT GCT GG | |  |  |  |
| Bif164F | Bifidobacteria (164-181) | GGGTGGTAATGCCGGATG | | | 62°C | [[48](#_ENREF_48)] |
| Bif662R | Bifidobacteria (662-679) | CCACCGTTACACCGGGAA | | |  |  |
| qBac560F | Bacteroides (560-584) | TTTATTGGGTTTAAAGGGAGCGTA | | | 50°C | [[49](#_ENREF_49)] |
| qBac725R | Bacteroides (725-646) | CAATCGGAGTTCTTCGTGATATCTA | | |  |  |

^*^Bases numbered relative to *E. coli* 16S rRNA sequence.

**GC clamp added to the 5’ end of the primer, 5’ CGC CCG CCG CGC GCG GCG GGC GGG GCG GGG GCA CGG GGG G 3’
