# Supplementary material for: Dietary Fat Content and Fiber Type Modulate Hind Gut Microbial Community and Metabolic Markers in the Pig
Source: PLoS One. 2013 Apr 3;8(4):e59581. doi: 10.1371/journal.pone.0059581 (PMC3616062; doi:10.1371/journal.pone.0059581)
Supplement: Table S3 — Primers for fat oxidation and inflammatory genes for Real-time PCR. (DOCX) [file pone.0059581.s003.docx]

**Table 3S**. Primers for fat oxidation and inflammatory genes for Real-time PCR

| Gene | Forward | Reverse |
| --- | --- | --- |
| 18S | 5'-ATC CCT GAG AAG TTC CAG CA-3' | 5'-CCT CCT GGT GAG GTC GAT GT-3' |
| GAPDH | 5'-GGG CAT GAA CCA TGA GAA GT-3' | 5'-TGT GGT CAT GAG TCC TTC CA-3' |
| TNFα | 5'-CGT CGC CCA CGT TGT AGC CAA T-3' | 5'-GCC CAT CTG TCG GCA CCA CC-3' |
| IL6 | 5'-TCT GGG TTC AAT CAG GAG ACC TGC-3' | 5'-TGC ACG GCC TCG ACA TTT CCC-3' |
| PGC1α | 5'-TAA AGA TGC CGC CTC TGA CT-3' | 5'-TGA CCG AAG TGC TTG TTC AG-3' |
| PPARα | 5'-TGC CAG TAT TGT CGT TTC CA-3' | 5'-GGC CTT GAC CTT GTT CAT GT-3' |
| ACO | 5'- GGA CGG CAG TCC AGA GAA TA-3' | 5'-GGT GGC GCT CTT CTT AAC AG-3' |
| CPT1α | 5'-GTC AGC GTA GCA AGT GGA CA-3' | 5'-GTG ACG TTA CAT CCC CTG CT-3' |
| FAS | 5'-AGT TTG TGA TGG AGA ACA CGG CCT-3' | 5'-TGT TCA CAC GTG GTG CAA GGG TTA-3' |
| SREBP-1c | 5'-ACC GCT CTT CCA TCA ATG AC-3' | 5'-AAT GTA GTC GAT GGC CTT GC-3' |
