# Supplementary material for: Dietary Fat Content and Fiber Type Modulate Hind Gut Microbial Community and Metabolic Markers in the Pig
Source: PLoS One. 2013 Apr 3;8(4):e59581. doi: 10.1371/journal.pone.0059581 (PMC3616062; doi:10.1371/journal.pone.0059581)
Supplement: Table S4 — Effect of different fiber types and fat content on gene expression in intestinal sections. (DOCX) [file pone.0059581.s004.docx]

**Table 4S**. Effect of different fiber types and fat content on gene expression in intestinal sections

|  |  | **LFD** | | **HFD** | |  | **Fat level** | | **Fiber** | | **P-value** | | |
| --- | --- | --- | --- | --- | --- | --- | --- | --- | --- | --- | --- | --- | --- |
| **Section** | **Gene** | **Inu** | **Sol** | **Inu** | **Sol** | **SE** | **LFD** | **HFD** | **Inu** | **Sol** | **Fat** | **Fiber** | **Fat*Fiber** |
| Cecum | IL6 | 0.78 | 1.08 | 0.85 | 1.18 | 0.17 | 0.93 | 1.02 | 0.81^b^ | 1.13^a^ | 0.61 | 0.07 | 0.90 |
|  | PGC1α | 1.30 | 1.00 | 1.35 | 1.64 | 0.33 | 1.15 | 1.49 | 1.32 | 1.32 | 0.33 | 0.99 | 0.40 |
|  | TNFα | 1.04 | 0.95 | 1.20 | 1.10 | 0.16 | 0.99 | 1.15 | 1.12 | 1.03 | 0.38 | 0.61 | 0.97 |
| Ileum | IL6 | 1.77 | 1.38 | 1.44 | 2.10 | 0.55 | 1.58 | 1.77 | 1.61 | 1.74 | 0.74 | 0.82 | 0.37 |
|  | PGC1α | 1.35 | 1.34 | 1.48 | 1.41 | 0.43 | 1.34 | 1.44 | 1.36 | 1.64 | 0.83 | 0.38 | 0.94 |
|  | TNFα | 1.45 | 1.07 | 1.36 | 1.32 | 0.35 | 1.26 | 1.34 | 1.40 | 1.20 | 0.84 | 0.57 | 0.85 |
| Jejunum | IL6 | 2.55^a^ | 0.66^b^ | 2.21^a^ | 1.82^a^ | 0.66 | 1.61 | 2.01 | 2.38^a^ | 1.24^b^ | 0.14 | 0.03 | 0.12 |
|  | PGC1α | 1.08 | 1.46 | 1.19 | 0.90 | 0.21 | 1.27 | 1.05 | 1.14 | 1.18 | 0.82 | 0.26 | 0.09 |
|  | TNFα | 1.78^a^ | 1.11^ab^ | 1.83^a^ | 0.56^b^ | 0.56 | 1.45 | 1.20 | 1.81^a^ | 0.84^b^ | 0.32 | 0.06 | 0.33 |
| Duodenum | IL6 | 1.23 | 2.39 | 1.57 | 1.21 | 0.64 | 1.81 | 1.39 | 1.40 | 1.80 | 0.83 | 0.73 | 0.75 |
|  | PGC1α | 0.44 | 0.52 | 0.51 | 0.38 | 0.16 | 0.48 | 0.44 | 0.48 | 0.45 | 0.80 | 0.88 | 0.53 |
|  | TNFα | 0.85 | 1.27 | 1.24 | 1.32 | 0.22 | 1.06 | 1.28 | 1.05 | 1.30 | 0.35 | 0.29 | 0.46 |

LFD, low fat diet-fed pigs; HFD, high fat diet-fed pigs; Inu, inulin diet-fed pigs; Sol, solka floc diet fed pigs. Data are presented as least-square means ± SE. Different letters within rows indicate significant difference (P < 0.05)
